# Increased Expression of GLP-1R in Proliferating Islets of Men1 Mice is Detectable by [^68^Ga]Ga-DO3A-VS-Cys^40^-Exendin-4 /PET

**DOI:** 10.1038/s41598-017-18855-0

**Published:** 2018-01-15

**Authors:** Azita Monazzam, Joey Lau, Irina Velikyan, Su-Chen Li, Masoud Razmara, Ulrika Rosenström, Olof Eriksson, Britt Skogseid

**Affiliations:** 1 Department of Medical Sciences, Uppsala University, Uppsala, Sweden; 2 Department of Medical Cell Biology, Uppsala University, Uppsala, Sweden; 3 Department of Medicinal Chemistry, Uppsala University, Uppsala, Sweden

## Abstract

Multiple endocrine neoplasia type 1 (MEN1) is an endocrine tumor syndrome caused by heterozygous mutations in the MEN1 tumor suppressor gene. The MEN1 pancreas of the adolescent gene carrier frequently contain diffusely spread pre-neoplasias and microadenomas, progressing to macroscopic and potentially malignant pancreatic neuroendocrine tumors (P-NET), which represents the major death cause in MEN1. The unveiling of the molecular mechanism of P-NET which is not currently understood fully to allow the optimization of diagnostics and treatment. Glucagon-like peptide 1 (GLP-1) pathway is essential in islet regeneration, i.e. inhibition of β-cell apoptosis and enhancement of β-cell proliferation, yet involvement of GLP-1 in MEN1 related P-NET has not yet been demonstrated. The objective of this work was to investigate if normal sized islets of Men1 heterozygous mice have increased Glucagon-like peptide-1 receptor (GLP-1R) expression compared to wild type islets, and if this increase is detectable *in vivo* with positron emission tomography (PET) using [68Ga]Ga-DO3A-VS-Cys40-Exendin-4 (68Ga-Exendin-4). 68Ga-Exendin-4 showed potential for early lesion detection in MEN1 pancreas due to increased GLP1R expression.

## Introduction

One of the most common inherited genetic syndromes related to neuroendocrine tumors is multiple endocrine neoplasia type 1 (MEN 1). Heterozygous mutations in the MEN1 tumor suppressor gene is usually inherited (can also occur sporadically) and loss of the wild type allele through somatic mutations in specific organs (e.g. endocrine cells of the pancreas, parathyroid or pituitary gland) induces tumor formation. Pancreatic neuroendocrine tumors (P-NET) occur in more than 80% of MEN1 patients^1^ and is the major reason for MEN1-related death^2–4^. Treatment of these patients demands multidisciplinary care including surgery, chemotherapy and targeted therapies^5^. Radical surgery can potentially cure the patient, but in cases with metastases only palliative care is available^6,7^. Late detection results in a higher number of patients with metastatic disease^8^, thus early detection and appropriate selection of surgical candidates is critical for optimal management of P-NETs. The pancreas of the young MEN1-gene carrier typically consist of a number of pre-neoplasias and microadenomas^9^, which later may transform to malignant tumors. Knowledge of key factors involved in initiation of pancreatic endocrine neoplasms might be important for the development of new methods for early and accurate detection of these lesions.

Heterozygous Men1 mutant mice mimic the human MEN1 syndrome and develop multiple endocrine tumors, mainly in the pancreas, parathyroid, and less frequently in the adrenal gland. Ninety percent of Men1 heterozygous mice develop islet cell hyperplasia and adenomas at 20 months of age^10^. The proliferating endocrine cells^11^ in Men1 mice serve as a valuable model for studies of the pathophysiology and molecular events of importance for initiation of tumorigenesis in MEN1 P-NETs using preclinical *in vitro*, *ex vivo* and *in vivo* methods.

GLP-1 pathway regulating inhibition of β-cell apoptosis^12^ and stimulation of β-cell proliferation is important in islet regeneration^13,14^. It has also been shown that the proliferative effect of GLP-1 in pancreatic islets synergistically increases when menin is inhibited^15^. The expression of the GLP-1 receptor (GLP-1R) has been studied in a number of clinical positron emission tomography (PET) trials of patients with P-NETs. In some of these trials cases of MEN1 associated P-NETs were included wherein Exendin-4 imaging shown potential for detection and localization of benign insulinoma^16^ but data on malignant insulinomas and glucagonomas are more ambiguous^17,18^. To clarify if GLP1 pathway is involved in initiation of proliferation in MEN1 pancreatic neoplasm and if GLP-1R expression could reflect this transformation^19^, we performed quantitative PCR as well as ^68^Ga-Exendin-4/PET-studies in Men1 mice.

## Materials and Methods

### Radiochemistry

The ^68^Ge/^68^Ga generator (IGG101, Eckert & Ziegler) was eluted with 0.1 N HCl collecting top fraction of 3.5 mL. The pH of the eluate was adjusted using acetate buffer to 4.6–5.0, and then the precursor DO3A-VS-Cys^40^-Exendin-4 (synthesized as previously described^20^ was added (10 nmol). EtOH (10–20% volume) was added to the reaction mixture to suppress radiolysis and formation of radioactive by-products. After the labeling reaction at 75 °C for 15 minutes the product was purified on a solid phase extraction cartridge (C-8, Waters or HLB, Oasis) to assure elimination of possible radioactive impurities, and it was eluted with 1 ml of 50% ethanol, and thereafter diluted with phosphate buffer for physiological pH and tonicity. A sample was taken for determination of radiochemical purity, peptide concentration, and pH. The total radioactivity of the product was then measured in an ionization chamber.

The quality control on radiochemical purity and determination of the concentration of the peptide was conducted using high pressure liquid chromatography (LaChrom, Hitachi, VWR). The dual detection was performed using sequentially coupled UV- (L-2400) and radiation detectors (Bioscan). The separation of the analyte from the impurities was accomplished using reversed phase analytical column (Discovery BIO Wide Pore C5; Sigma-Aldrich) with covalently bonded pentylsilane. Mobile phases consisted of 10 mM trifluoroacetic acid for A and 70% acetonitrile, 30% H_2_O, and 10 mM trifluoroacetic acid for B. Gradient elution was conducted allowing 35% B for 0–2 min followed by increase of B phase from 35% to 100% within 2–9 min. The fraction of B was kept at 100% during 9–12 min. The UV detection was conducted at 220 nm, and flow rate was 2.0 mL/min. EZChrom Elite Software Package (Agilent Technologies) was used for the data acquisition and processing.

### Animal model

The Men1 mouse is a conventional heterozygous knockout mouse, which was a kind gift by Professor Hayward of the Queensland Institute of Medical Research, Herston, Australia. Deletion of the second exon of the MEN1 gene produces heterozygous non-sense mutation of menin which leads to development of pancreatic hyperplasia and adenomas in 90% of animals at the age of 20 months. In this study we utilized 10 and 20 month old Men1 mice. Wild type littermates served as control. Maintenance of animals as well as experimental design was in agreement with the Swedish animal protection legislation and European regulations, and were approved by the animal ethics committee (Uppsala djurförsöksetiska nämnd) in Uppsala, Sweden (permit number: C187/14).

### ^68^Ga-Exendin-4 biodistribution

The biodistribution studies were performed one hour post injection of 0.2–0.8 MBq ^68^Ga-Exendin-4 (corresponding to 2.9–4.0 µg/kg peptide dose) in heterozygous and wild type mice (four mice per group). The mice were euthanized and dissected, and organs of interest were collected and weighed. Radioactivity in the tissue was measured in an automated gamma counter and normalized to Disintegrations Per Minute **(**DPM)/mg tissue and a tissue-to-blood ratio was calculated.

### *Ex vivo* Autoradiography

To achieve high resolution imaging and quantification of ^68^Ga-Exendin-4 in endocrine pancreas, *ex vivo* autoradiography^21^ of pancreas was performed on heterozygous and wild type mice (four mice per group).

Mice were injected with 0.2–0.8 MBq ^68^Ga-Exendin-4 (corresponding to 2.9–4.0 µg/kg peptide massdose) in the tail vein. One hour post injection, the mice were euthanized, and the pancreas was excised, immediately frozen and sectioned. Three consecutives cryosections (20 μm thick) were prepared, shortly air-dried, and apposed to phosphor imaging plates for 2 h. The exposed imaging plates were scanned in a FUJI BAS 5000 reader (Phosphorimager 445 SI, Amersham Biosciences, Uppsala, Sweden). The resulting scanned images were processed using ImageJ 1.48 v software by first defining a region of interest (ROI) of the entire tissue section. Then ROIs of the regions with high radioactive signal intensity, were generated to define the islets and it was confirmed with IHC. Radioactive signal in the exocrine pancreas was calculated by subtraction of the total values of all islets from the value of the whole section. Background values for the overall phosphor image plate were also subtracted from all data samples. The mean values of the radioactive signals for the islets and the exocrine pancreas were presented as a ratio of photostimulated luminescence (PSL/mm^2^) for each section^21^.

### Immunohistochemistry

In some PET *ex vivo* autoradiography experiments, the same sections (n = 10) of the pancreas as used for the autoradiography (n = 24) were stained with GLP1-R antibody (abcam, Cambridge, UK). In some other PET *ex vivo* autoradiography experiments, two adjacent sections (6 μm) were stained with glucagon (n = 10) and insulin (n = 10) antibodies (abcam, Cambridge, UK). Sections were fixed in pre-cooled (−20 °C) acetone for 10 min. After evaporation of acetone from the tissue sections for 20 min at room temperature, the slides were washed in phosphate buffered saline (PBS) 2 times (5 min each), incubated in 0.3% hydrogen peroxide solution at room temperature for 10 min to block endogenous peroxidase activity, washed in PBS, followed by incubation with the primary GLP-1R (ab39072), insulin (ab63820) or glucagon antibody (ab92517). The slides were washed and incubated in Flex+/HRP (Dako, Glostrup, Denmark) for 15 minutes. After another wash, the slides were developed with DAB+ (Dako, Glostrup, Denmark), counterstained with hematoxylin (Biocare Medical, San Diego, CA, USA), rinsed in water, dehydrated, and finally mounted.

### PET/MRI imaging

One wild type and one heterozygous 20 month old mouse were subjected to PET/MRI-imaging. One hour post injection of ^68^Ga-Exendin-4 (∼0.5 MBq), the animals were euthanized, the both kidneys were extirpated and the animals were positioned in the supine position in the scanner gantry of the animal PET- 3T MR scanner (nanoScan, Mediso, Medical Imaging Systems, Budapest, Hungary) and examined by whole body PET for one hour in list mode followed by a MR examinations. PET data were reconstructed into a static image using a Tera tomo 3D algorithm (4 iterations, voxel size: 0.40 mm, matrix: 255 × 255). MRI measurements were performed at 3T using a whole body transmit-receiver coil. T_1_-weighted spin echo (T1W SE) and T_2_-weighted fast spin echo (T2W FSE) images were acquired in the axial and coronal planes. The main acquisition parameters of axial images were field of view (FOV) 40 × 40 mm^2^, acquisition matrix 384 × 192, spatial resolution 0.1 × 0.21 mm^2^, slice thickness 1 mm, interslice gap 0.1 mm. T1W SE images were measured with repetition (TR) and echo time (TE) 360 and 13 ms, respectively. Other parameters were as follows: bandwidth (BW), 40 000 Hz; number of signal averages (NSA), 4; scan durations, 4 min 50 sec. Axial T2W FSE were acquired with: TR/TE, 3000/60; BW, 60 000 Hz; NSA, 12; scan duration 14 min 50 sec. The spatial parameters of coronal T1W SE and T2W FSE images were as follows: FOV, 50 × 50 mm^2^; acquisition matrix 384 × 192, spatial resolution 0.13 × 0.26 mm^2^, slice thickness 1 mm, gap 0.1 mm. Coronal T1W SE were acquired with: TR/TE, 360/13; BW, 40 000 Hz; NSA, 4; scan duration 4 min 50 sec. Coronal T2W FSE measurement parameters were as follows: TR/TE, 3000/60; BW, 60 000 Hz; NSA, 8; scan duration 9 min 56 sec.

PET and MRI images were then processed using PMOD v3.510 (PMOD Technologies Ltd, Zurich, Switzerland)”.

### Isolation of endocrine pancreas, RNA preparation and Quantitative real time PCR

Pancreatic islets of heterozygous and wild type mice (six per group) were isolated using collagenase digestion and density gradient purification^22^. Ten mg Collagenase A (Clostridium histolyticum; Roche Diagnostics, Mannheim, Germany) dissolved in 4 mL Hanks’ buffer (National Bacteriological Laboratory, Stockholm, Sweden) was injected into the common bile duct (CBD) of a euthanized animal. The pancreas was then removed and digested at 37 °C. Intact islets were separated by a density gradient (Histopaque-1077, Sigma-Aldrich, St. Louis, MO, USA), washed, handpicked and transferred to lysis buffer for qPCR analysis^23^.

RNAqueous Micro Kit (Ambion, Life Technologies, USA) was used to isolate total RNA from pancreatic islets according to the manufacturer’s recommendations. The purified RNA was eluted with RNase-free water (Life Technologies) and the concentration was determinated using NanoDrop 1000 (NanoDrop, Wilmington, DE, USA). One µg of total RNA from pancreatic islets was reverse transcribed and converted to cDNA by the iScript cDNA synthesis Kit (Bio-Rad, Hercules, CA, USA). The cDNA was used to analyze glucagon, GLP-1R, insulin I, insulin II, and MEN1 gene expression by using primers described in Table 1. Mice have two functional insulin genes located on separate chromosomes; insulin I and insulin II. Ins1 arose from a duplication of the insulin II inherited gene ∼20 million years ago and has since been retained in the mouse genome^24^. Quantitative real time PCR (QRT-PCR) reaction was performed on 20 µL, and each reaction included 10 ng of cDNA, 2× SsoAdvanced Universal SYBR Green Supermix (Bio-Rad) and, 500 nM forward and reverse primers. An initial amplification was done with a denaturation step at 95 °C for 10 min, followed by 40 cycles of denaturation at 94 °C for 15 seconds, and primer annealing and extension at 60 °C for 1 minute using the Stratagene Mx3005P real time PCR System (Agilent Technologies, Waldbronn, Germany). The data were evaluated by the 2−∆∆CT method^25^ using the mRNA level of HPRT (set to 1).

**Table 1:** Primer sequences used in QRT-PCR analysis.

Symbol	Description	Primer Sequences	Product (bp)
*GCG*	Glucagon	F: 5′-GGCACATTCACCAGCGACTACA-3′	174
		R: 5′-GCCCTCCAAGTAAGAACTCACATC-3′	
*GLP1R*	glucagon-like peptide 1 receptor	F: 5′-TCAGAGACGGTGCAGAAATG-3′	183
		R: 5′-CAGCTGACATTCACGAAGGA-3′	
*HPRT*	Hypoxanthine guanine phosphoribosyl transferase	F: 5′-CGTCGTGATTAGCGATAGTG-3′	178
		R: 5′-ACAGAGGGCCACAATGTGAT-3′	
*INS1*	insulin I	F: 5′-CCTTAGTGACCAGCTATAATCAGAG-3′	248
		R: 5′-CACTTGTGGGTCCTCCACTT-3′	
*INS2*	insulin II	F: 5′-GAAGTGGAGGACCCACAAGTG-3′	112
		R: 5′-GATCTACAATGCCACGCTTC-3′	

### Statistical analysis

All calculations were performed using GraphPad Prism version 6 (San Diego, CA, USA). All values are expressed as mean ± SEM. Probabilities (*P*) of chance differences between the groups were calculated with multiple t-test (biodistribution studies) and unpaired t-test (*ex vivo* autoradiography and qPCR studies), where differences at the 95% confidence level (*P* < 0.05) were considered significant.

## Results

### ^68^Ga-Exendin-4 biodistribution

**Figure 1:**
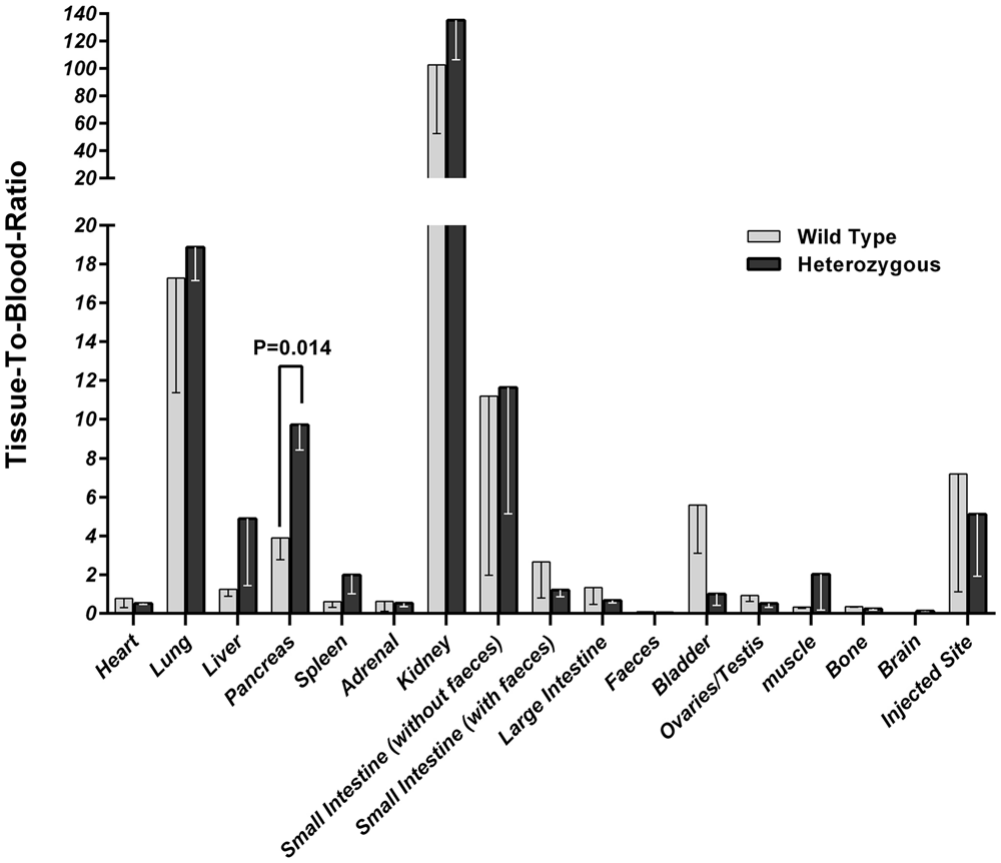
Comparative organ distribution of ^68^Ga-Exendin-4 in heterozygous and wild type 20 months old mice.

Biodistribution of ^68^Ga-Exendin-4 at 60 min after intravenous injection in heterozygous and wild type 20 month old mice is shown in Fig. 1. Radioactivity accumulated in pancreas of heterozygous mice was 60% higher than that in wild type (*P* < 0.01) and such difference was not observed in any other organ. Although the pancreas uptake of ^68^Ga-Exendin-4 was high, concurrent pronounced accumulation in kidney and lung may result in a low pancreas-to-background ratio of tracer in rodents. In biodistribution study of 10 month old mice, there was no significant difference between heterozygous and wild type littermates in accumulation of the tracer in any vital organ.

### *Ex vivo* Autoradiography and Immunohistochemistry

**Figure 2:**
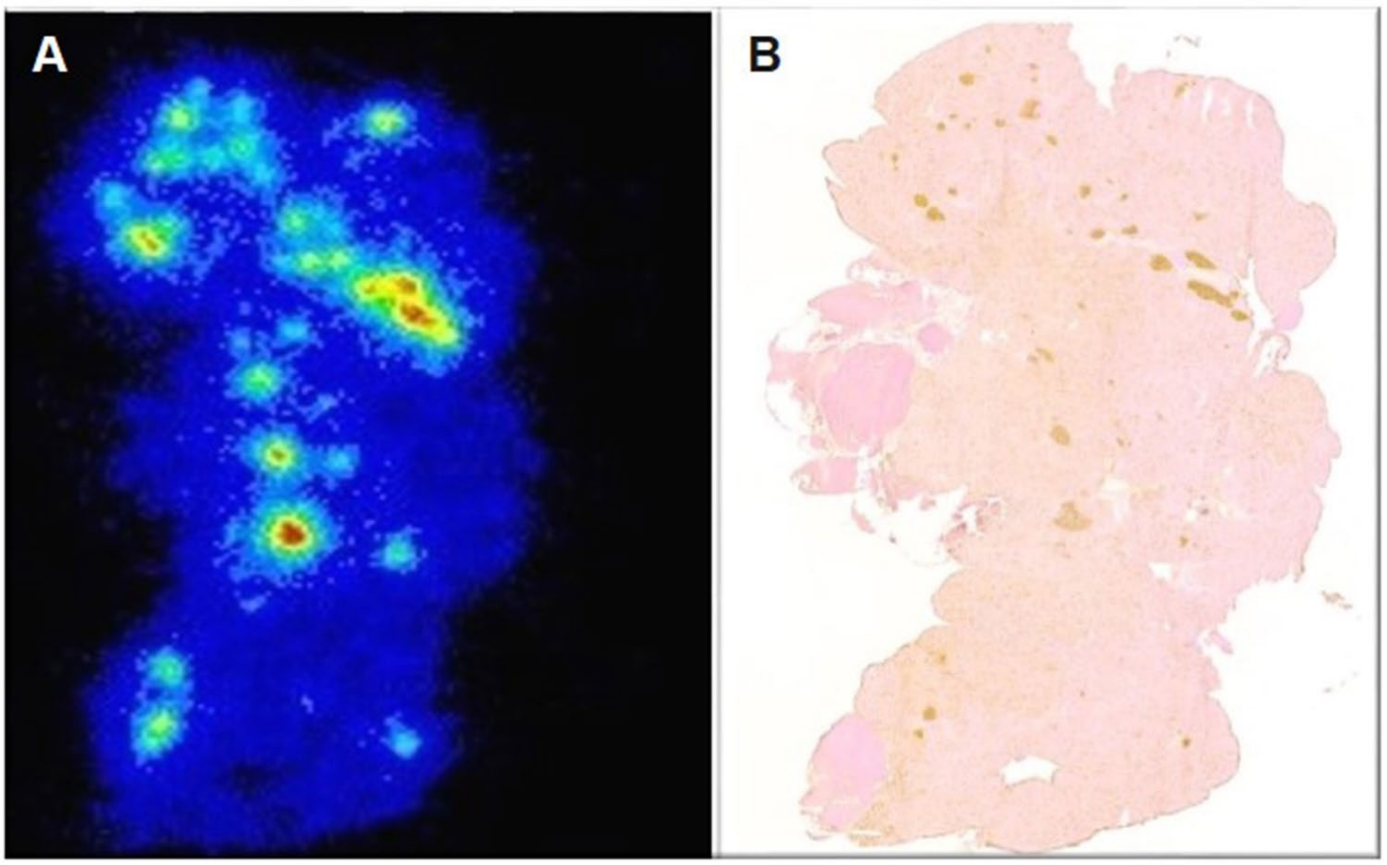
Co-localization of autoradiography ^68^Ga-Exendin-4 signals (**A**), and GLP1-R immunohistochemistry staining spots (**B**) in the same pancreatic section from a 20 months old heterozygous mouse.

**Figure 3:**
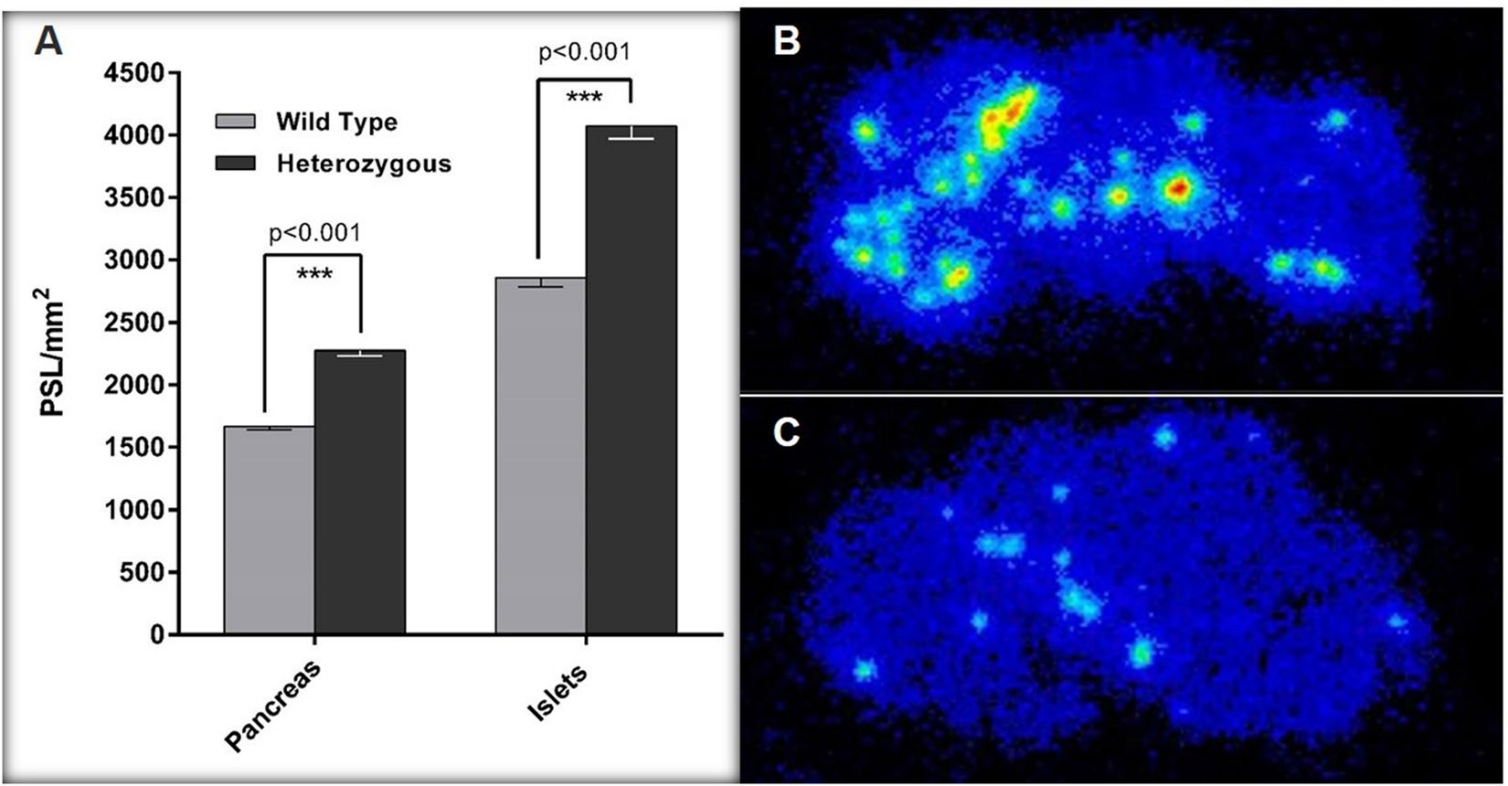
(**A**) Quantitative *ex vivo* autoradiography GLP-1R expression in total and endocrine pancreas of heterozygous vs. wild type 20 months old mice. (**B**) Representative ex vivo PET autoradiography using ^68^Ga-Exendin-4 in pancreas of 20 months heterozygous vs (**C**) wild type mouse injected a similar amount of radioactivity.

The *ex vivo* autoradiography revealed focal ^68^Ga-Exendin-4 uptake in multiple areas throughout the sections, likely corresponding the radiotracer accumulation in the islets. Consecutive GLP1-R immunostaining carried out on the same section displayed a similar staining pattern and confirmed that the localizations of intense radioactive uptake certainly matched the distribution of pancreatic islets (Fig. 2). As displayed in Fig. 3, there was a significant increase in tracer uptake in total pancreas (37%) as well as the islets of pancreas (42%) in heterozygous 20 month old mice compared to total pancreas and islets of wild type littermates. In concordance with biodistribution study, there was no difference in the tracer accumulation in islets in 10 month old heterozygous and wild type mice.

**Figure 4:**
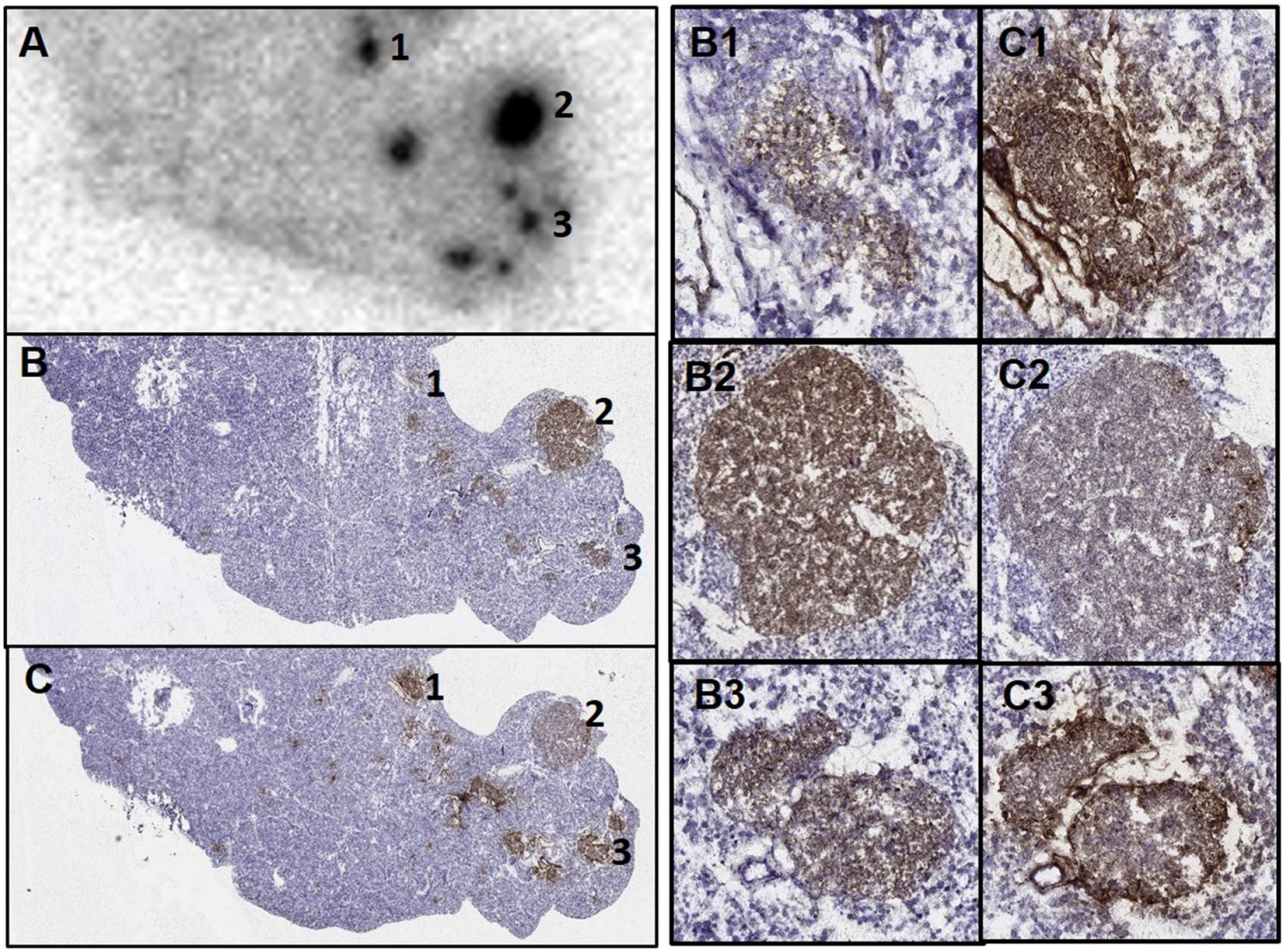
After *ex-vivo* ^68^Ga-Exendin-4 autoradiography (**A**), two adjacent pancreas sections were stained for insulin (**B**) or glucagon (**C**). Three different hormonal profiles were recognized in the pancreatic lesion with ^68^Ga-Exendin-4: low expression of insulin (B1) and high expression of glucagon (C1); high insulin (B2) and low glucagon (C2); and normal expression of both insulin (B3) and glucagon (C3).

By comparing ^68^Ga-Exendin-4 *ex vivo* autoradiography images with glucagon and insulin staining of the consecutive sections of the pancreas from heterozygous 20 month old mice, indications were found that GLP-1R is overexpressed in microadenomas; the same GLP-1R expression pattern was observed both in lesions with strong glucagon immunoreactivity and weak insulin expression, as well as in lesions predominantly insulin immunoreactive (Fig. 4).

### PET/MRI imaging

**Figure 5:**
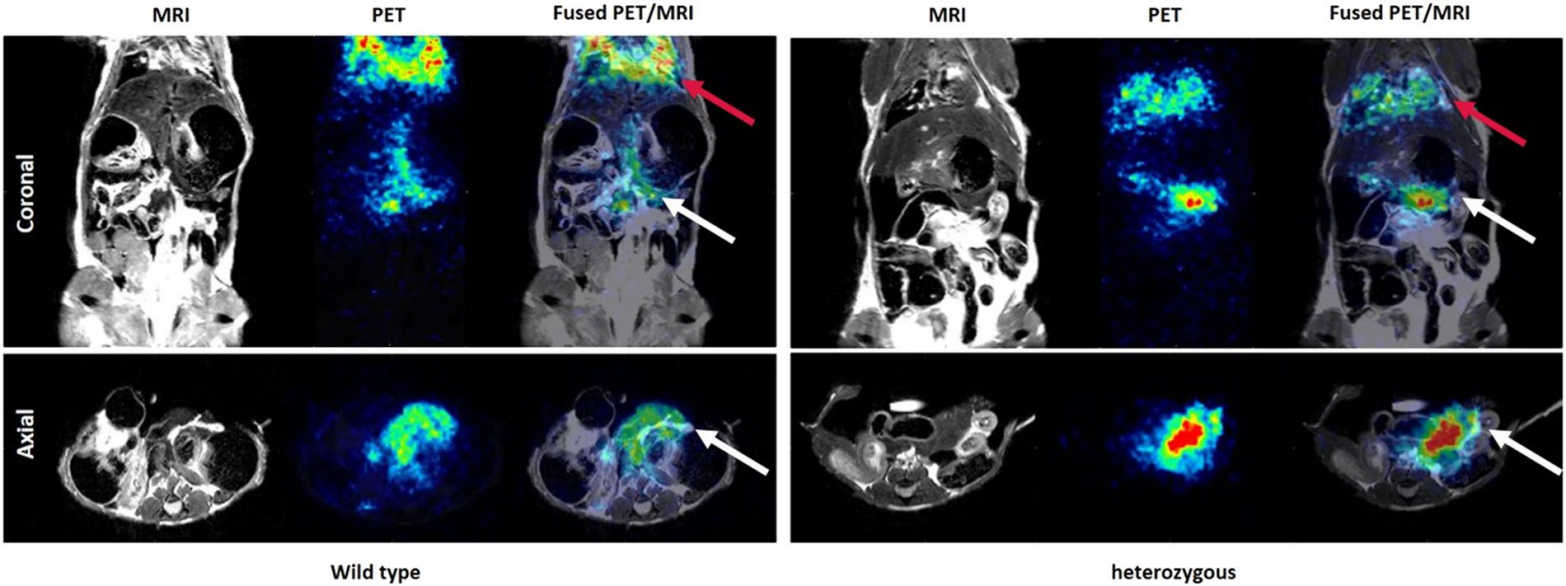
Coronal and axial PET, MRI and fusion images of 68Ga-Exendin-4 in wild type and heterozygous 20 months old mice (red arrows: lungs, white arrows: pancreas).

In PET/MRI imaging of ^68^Ga-Exendin-4, due to bilateral nephrectomy and no interfering signal from kidneys, the tracer uptake in the pancreas could be visualized. In compliance with *ex vivo* autoradiography, a higher tracer uptake in pancreas of heterozygous 20 month old mice compared to wild type littermates was observed (Fig. 5).

### Quantitative PCR

**Figure 6:**
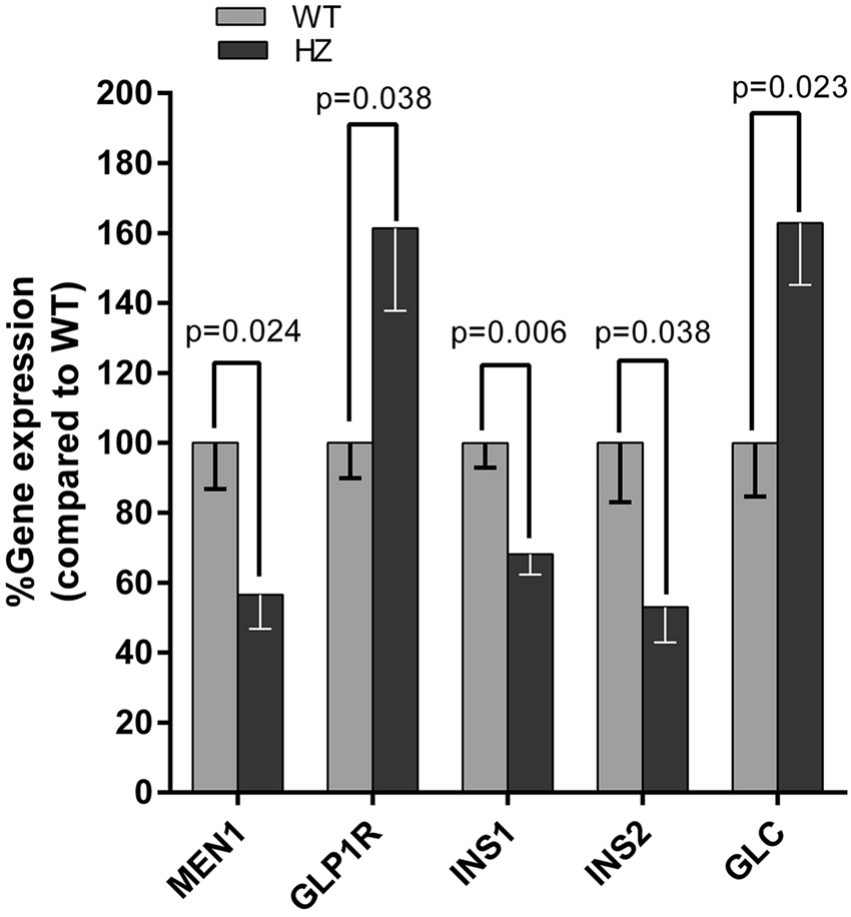
Relative MEN1, GLP-1R, insulin I (INS1), insulin II (INS2) and glucagon (GLC) mRNA expression measured by qPCR in islets of heterozygous and wild type 20 months old mice.

The GLP-1R gene expression level was 61% higher while MEN1 gene expression was 43% lower in islets isolated from heterozygous 20 month old mice compared to that of age-matched wild type (Fig. 6). Interestingly, the mRNA expressions of insulin I and insulin II were lower, 32% and 47% respectively, and glucagon was 63% higher in heterozygous islets. The qPCR result indicated that the GLP1R upregulation in MEN1 islets was not solely associated with insulin production. However, there was no significant difference in GLP1-R gene expression in islets of 10 month old heterozygous mice compared to wildtype littermates (Supplementary Figure 2).

## Discussion

The clinical recognition of MEN1 related P-NETs are generally correlated to their abnormal hormone secretion. However, hormonal syndromes often occur late and indicate presence of metastases in more than 50% of the patients^26^. Early detection of initial transformation in endocrine pancreas of MEN1 gene carriers would be beneficial for stratification of high-risk patients. Noninvasive PET imaging of molecular markers in oncology offers earlier diagnosis, guide to treatment selection, early treatment follow-up, and personalized treatment^27,28^. Yet, most commonly, PET tracers are utilized during advanced tumor stages and for treatment follow up. Our research group is interested in assessing the potential use of PET to detect early stage in tumor development. In addition, a sensitive PET-tracer for early tumor progression might be of value in preclinical drug development studies for in vivo assessment of outcome.

Current PET tracers utilized in P-NET diagnostics are: ^18^F-FDG in poorly-differentiated neoplasms with high proliferative activity and loss of neuroendocrine features e.g. G3 PNET^29–31^; ^68^Ga-labelled somatostatin analogues in somatostatin receptor expressing P-NETs^32^; the amine precursors, ^18^F-DOPA and ^11^C-5-HTP, in functional P-NETs^33^ but is less sensitive in detection of low differentiated and nonfunctional tumors; and Exendin-4 labelled with various radioisotopes in benign insulinoma^34,35^. Our interest was to introduce a PET tracer for the detection of early pancreatic neoplasms in MEN1 patients. Due to the involvement of GLP1 pathway in proliferation of β-cells^36^, ^68^Ga-Exendin-4 was a logical choice to investigate if GLP-1R reflects the increased proliferation in islets of Men1 mice^11^.

^68^Ga-Exendin-4 has previously shown strong specific binding to the GLP-1R^37^. Competition studies in mouse demonstrated that the ^68^Ga-Exendin-4 uptake in lung and pancreas was GLP-1R mediated^38^. However, the high accumulation and retention of the tracer in kidneys make the accurate quantification of the tracer uptake in the pancreas of a mouse challenging. The potential solution has been demonstrated for other Exendin analogues using such inhibitors of renal uptake as albumin fragments and gelatin-based plasma expanders^39,40^. Another ^18^F-labeled Exendin-4 analogue demonstrated specific uptake in pancreatic islets with rapid renal clearance^41^. It is important to point out that this is a rodent model-specific technical issue. In human the distance between pancreas and left kidney is large enough to enable clear pancreatic visualization by PET without renal spill-over^42^. The kidney radiotracer retention will therefore not obscure ^68^Ga-Exendin-4 imaging of MEN1 related P-NETs in clinical studies.

Another limitation is the difficulties posed by the anatomical features of pancreas in mice, as well as the physical limits of PET imaging that requires image processing^43^. In our study, to overcome these obstacles, we performed ex-vivo PET autoradiography.

In this study we showed that proliferation in pancreatic islets reflects on amplified GLP1-R expression in heterozygous mice at the age of 20 months, when 90% of the animals develops adenomas. This conclusion was confirmed when no alteration was observed in islets of 10 month old heterozygous mice. This study also revealed that ^68^Ga-Exendin-4 displayed high sensitivity for detection of pancreatic islets or pre-neoplasias of Men1 mice which made the tracer attractive for noninvasive imaging for monitoring of disease progression. Overexpression of the GLP-1R due to MEN1 gene deficiency was confirmed with gene expression study.

So far the application of Exendin-4 imaging in P-NETs has been focused on detection of insulinoma but our study indicates that ^68^Ga-Exendin-4/PET imaging reflects the initiation of tumorigenesis that is characteristic of MEN1^9^. Our qPCR result of glucagon gene overexpression in islets of Men1 mice is in accordance with characteristics of microadenomas in MEN1 patients^44^.

Different level of glucagon and insulin immunoreactivity in MEN1 islets/microadenomas detected by ^68^Ga-Exendin-4/PET shows that GLP-1R overexpression in MEN1 islets is related to the increased proliferation regardless their hormonal characteristics. GLP-1R overexpression might persist^16,42^ or fade gradually^17,18^ during later tumor progression. The role of ^68^Ga-Exendin-4/PET in management of MEN1 gene carriers remains to be established and clinical studies are warranted in order to investigate if this tracer can identify individuals with increased islet proliferation that might benefit from early treatment institution perhaps prophylactic treatment before tumor occurrence. In conclusion, ^68^Ga-Exendin-4/PET demonstrated potential for lesion detection in MEN1 pancreas due to increased GLP1R expression in early tumorigenesis.

## Electronic supplementary material

Supplementary Information
