# Supplementary material for: Increased Expression of GLP-1R in Proliferating Islets of Men1 Mice is Detectable by [68Ga]Ga-DO3A-VS-Cys40-Exendin-4 /PET
Source: Sci Rep. 2018 Jan 15;8:748. doi: 10.1038/s41598-017-18855-0 (PMC5768696; doi:10.1038/s41598-017-18855-0)
Supplement: Supplementary file 1 — Supplementary Information [file 41598_2017_18855_MOESM1_ESM.pdf]

Supplementary Figures

Increased Expression of GLP-1R in Proliferating Islets of Men1 Mice is Detectable by [^{68}Ga]Ga-DO3A-VS-Cys^{40}-Exendin-4 /PET

Azita Monazzam, Joey Lau, Irina Velikyan, Su-Chen Li, Masoud Razmara, Ulrika Rosenström, Olof Eriksson, Britt Skogseid

Correspondence to: azita.monazzamedsci.uu.se

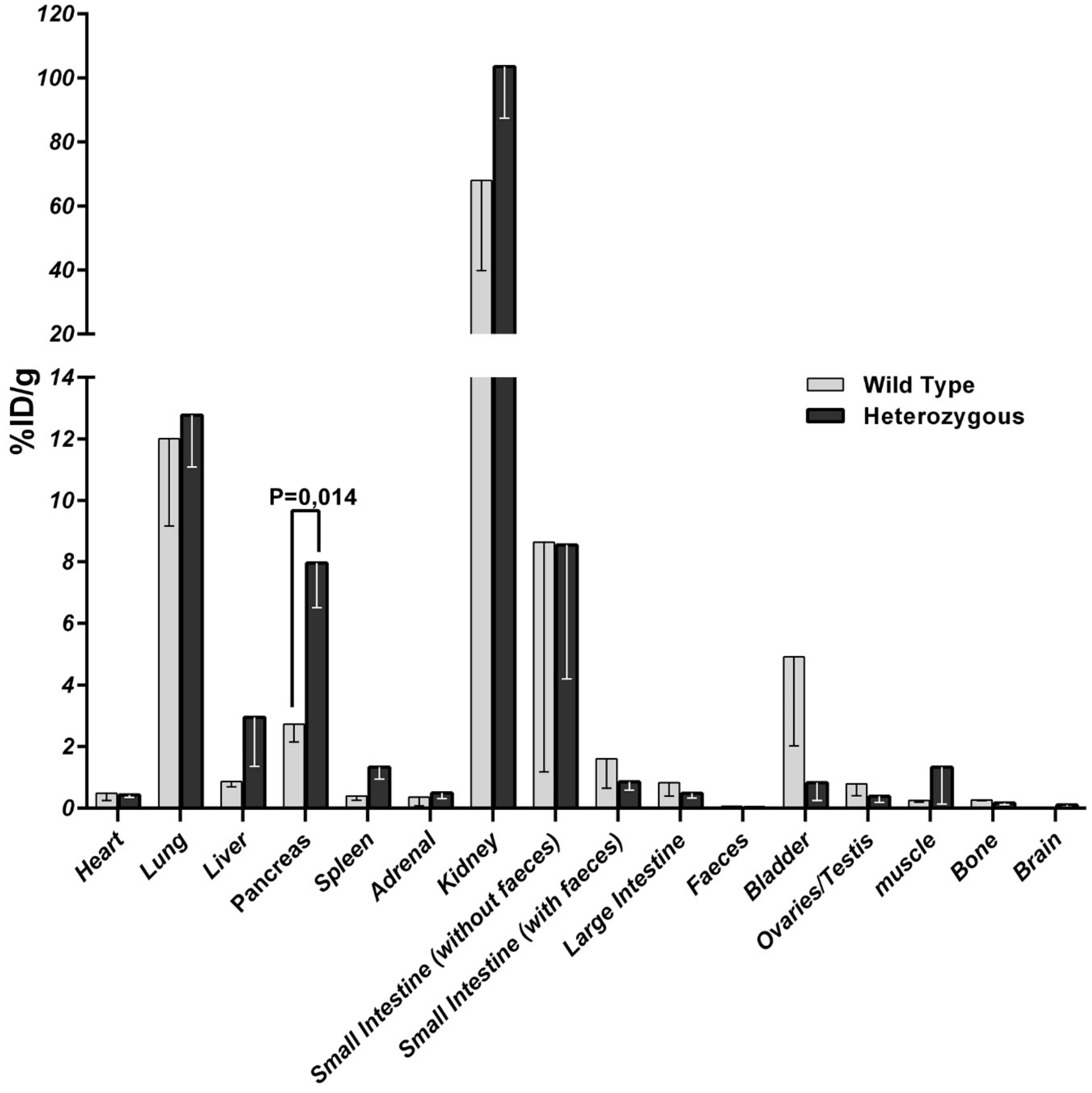

Supplementary figure 1 - organ distribution of 68Ga-Exendin-4 in heterozygous and wild type 20 months old mice. Values are presented as percentage of injected dose per gram of tissue (%ID/g) ± SD.

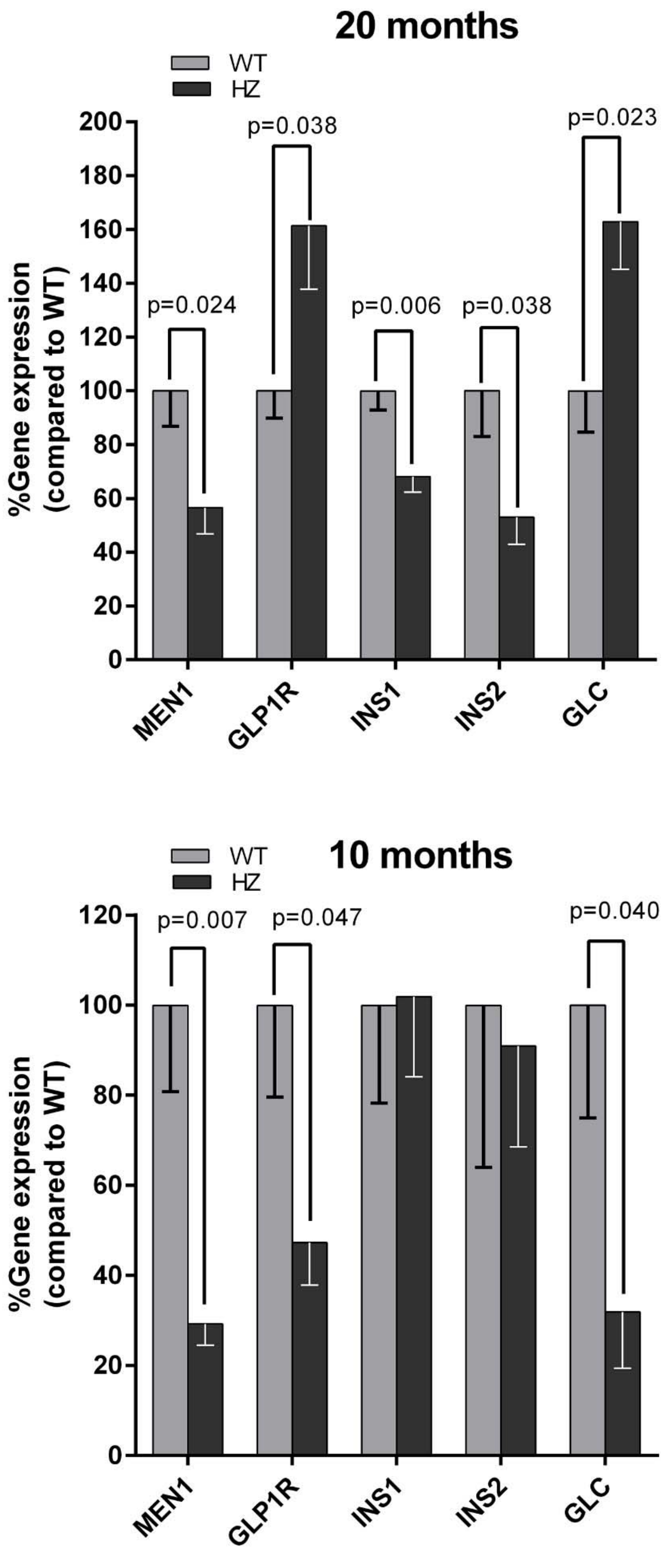

Supplementary figure 2 – Relative MEN1, GLP-1R, insulin I (INS1), insulin II (INS2) and glucagon (GLC) mRNA expression measured by qPCR in islets of heterozygous and wild type 10 and 20 months old mice.
